# Job satisfaction and job tenure of people with mental health disorders: a UK Biobank cohort study

**DOI:** 10.1177/14034948221119639

**Published:** 2022-08-25

**Authors:** Salahuddin Mohammad, Maud Miguet, Gull Rukh, Helgi B. Schiöth, Jessica Mwinyi

**Affiliations:** Department of Neuroscience, Uppsala University, Sweden

**Keywords:** Anxiety, depression, eating disorder, employment, job satisfaction, job tenure, mental illness, mood disorder, neuroticism, occupation, schizophrenia, stress disorder

## Abstract

**Aims:**

Job satisfaction plays an important role for the life quality and health of working individuals. While studies have shown that self-reported mental health conditions such as stress, anxiety and depression are associated with job satisfaction, a large population-based study exploring and comparing self-reported physician posed diagnosed conditions and their association with job satisfaction and job tenure is missing. This study addresses the gap along with exploring the impact of the neurotic personality trait and other possible contributing factors.

**Methods:**

Sixteen mental health disorders diagnosed by physicians, categorised into four major groups were investigated in relation to employment status (108,711 participants) and in relation to job satisfaction and job tenure (34,808 participants). Analyses were performed using linear regression adjusted for age, sex, townsend deprivation index, body mass index, education, physical activity, work hours and neuroticism.

**Results:**

Neurotic and stress disorders, eating disorders and other mental health disorders were strongly associated with lower job satisfaction and shorter job tenure in both unadjusted and adjusted analyses. Neuroticism was strongly linked to job satisfaction but was not associated with job tenure.

**Conclusions:**

**Study findings clarify the complex relationship of mental health with job satisfaction and job tenure, which is very important to understand in designing measures to improve working life participation of individuals with mental health issues.**

## Background

Job is an imperative part of people’s life in many aspects . The individual satisfaction with the job is important as it has an essential impact on health considering many types of diseases and work conditions [1, 2]. Mental health problems are common in industrialised countries and the integration of affected individuals in the job market is of utmost importance to combat their high unemployment rate [3]. Despite numerous supported employment programmes, individuals with mental health disorders show a high rate of job termination, with mental illness and job dissatisfaction as one of the main reasons [3–5].

Social scientists have been working for decades to construct a well-accepted definition of job satisfaction. Job satisfaction comprises the feelings of an employee towards the job with regard to motivation, content and positivity [1, 2], and there is growing evidence that the impact of job satisfaction on health is significant. A meta-analysis of 485 studies addressing job satisfaction, incorporating over 267,995 employees from different organisations in different parts of the world, explored the association between job satisfaction and physical and mental health [1]. The results of this meta-analysis suggested a positive and strong correlation of job satisfaction with mental health [1]. Similarly, another meta-analysis based on Hong Kong’s population, additionally investigated gender, culture and ethnic differences as moderators for job satisfaction and found that mental health was related to job satisfaction [2]. A recent life course perspective study conducted among American young adults starting at the age of 25 years and ending at the age of 39 years came to a similar result, showing that mental health is strongly related to job satisfaction trajectories [6]. Often the mental health of employees is assessed as part of the general health by questionnaires that focus mainly on self-reported complaints such as anxiety, depression, burn-out or emotional exhaustion, and has found job satisfaction to be low and strongly related with these conditions [1, 2]. Moreover, a recent study on 212 working adults recruited online shows that the highest rates of depression and stress were found in individuals with lower job satisfaction [7]. Individuals with lower job satisfaction are also more likely to be diagnosed with emotional problems [6]. Interestingly, people diagnosed with intellectual disability have been shown to experience higher job satisfaction compared with people without disability even with similar job satisfaction predictors [8], which may be relevant to consider in investigations that explore certain diagnoses that are connected with a higher likelihood for variances in the intellectual ability. However, as is widely acknowledged, the boundary between disorder and ‘normality’ in mental health conditions is not always clear [9], self-reported physician diagnosed conditions are more reliable in order to draw firm research conclusions.

While we observed a strong relationship between personality factors, individual mental health and job satisfaction [10], a recent meta-analysis reported that a neurotic personality trait is the strongest predictor among the personality traits in relation to job satisfaction [11]. Based on these observations, our group explored this aspect further and observed an independent causal association between neuroticism and job satisfaction [12]. Of note, a meta-analysis of 59 studies reported that high neuroticism provides a high risk for developing any common mental disorder [13]. This may be partly explained by the shared genetic influence among people with a mental health diagnosis and a higher rate of neurotic personality trait [14].

Concerning the higher unemployment rate in individuals with mental illnesses [3], many studies addressed job acquisition and rehabilitation to integrate affected individuals in the job market [3, 15–20]. Job retention has been found to be more difficult to achieve than acquisition [4]. Interestingly, investigations regarding job tenure focused mainly on employment programmes for individuals with mental disorders. These studies are mostly small scale studies and are focused on severe psychiatric conditions [3, 15–20].

Numerous studies have been conducted at an organisational level to study the mental health status of employees, relying on self-reported diagnoses of very prevalent disorders such as depression, anxiety or stress in specific occupations in relation to job satisfaction [1, 2]. Of note, no study has hitherto investigated job satisfaction in individuals with diagnosed mental health disorders. Moreover, studies on job tenure are very limited in sample size and address only severe mental illness in employment programmes [3, 15–20]. Large scale job tenure studies addressing both severe and non-severe mental health conditions in a broad range of different diagnosed mental health conditions among working individuals have not been conducted.

## Aims

The purpose of this study was to investigate and compare job satisfaction and job tenure among people with diagnosed mental health disorders in more than 150,000 participants of the UK Biobank. We also explored the impact of sociodemographic characteristics, work-related factors and the neurotic personality trait on job satisfaction and job tenure.

## Methods

### Study population: the UK Biobank

**Figure 1.**
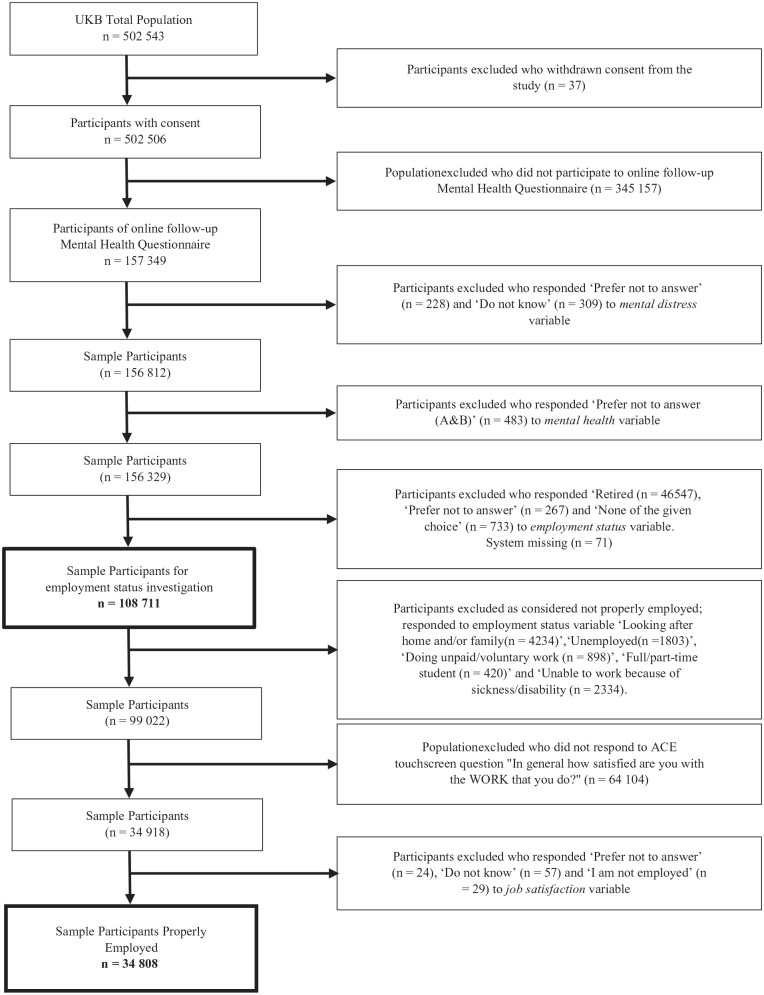
Flow chart of cohort.

In this study data from the UK Biobank were used, which is an open source prospective cohort covering information for over half a million participants and has been described previously in detail [21]. Briefly, adults aged 40–69 years were recruited between 2006 and 2010. Participants were registered with the National Health Service (NHS) in England, Wales or Scotland on their visit to a nearby assessment centre (within 25 miles of residence) on invitation. During the baseline assessment visit participants completed computer-assisted self-administered questionnaires, took part in face-to-face interviews, and also provided physical measures and biological samples [21]. In addition, individuals participated in follow-up and online base web questionnaires. Ethical approval for the UK Biobank study was granted from the NHS National Research Ethics Service North West (16/NW/0274) and the Regional Ethics Committee of Uppsala, Sweden further approved the data usage at our department. Written informed consents were taken from all participants with the right to withdraw at any time from the study. Of the total UK Biobank population (*N*=502,543), we excluded participants who ‘withdrew their consent for participation’; ‘did not participate to online follow-up mental health questionnaire’; answered ‘prefer not to answer’ or ‘do not know’ or ‘none of the given choices’ for mental distress question (field ID: 20499), mental health question (field ID: 20544) and employment status question (field ID: 6142). The remaining (*N*=108,711) participants were further scanned for information regarding job satisfaction and job tenure. Only people ‘in paid employment/self-employed’ referred to as ‘properly employed’ (*N*=34,808) were considered, as the participants falling into the categories ‘retired’, ‘looking after home and/or family’, ‘unemployed’, ‘doing unpaid/voluntary work’, ‘full/part-time student’ and ‘unable to work because of sickness/disability’ were excluded (Figure 1).

### Ascertainment of the variable

#### Job satisfaction and job tenure

Information on job satisfaction was collected by way of the UK Biobank touchscreen question: ‘in general how satisfied are you with the work that you do?’ (field ID: 4537) and treated as an ordinal variable. The options to respond were ‘extremely happy’, ‘very happy’, ‘moderately happy’, ‘moderately unhappy’, ‘very unhappy’, ‘extremely unhappy’, ‘do not know’, ‘prefer not to answer’ and ‘I am not employed’. The answers ‘do not know’, ‘prefer not to answer’, ‘I am not employed’ and ‘participant with missing data’ were considered as missing, and the final scale ranged from 1 to 6. A higher score corresponds to higher job satisfaction. Job tenure was defined as time (years) employed in the main job and explored with the question ‘how many years have you worked in your current job?’ (field ID: 757). In the case more than one ‘current job’, participants were asked to answer the question for the main job only, that is, ‘the job that you spend most of your time doing’. Higher values represent longer job tenure. The variables ‘do not know’, ‘prefer not to answer’ and ‘participant with missing data’ were considered as missing. The variables were assessed using baseline data collected during the years 2006–2010 and covered with the highest number of participants compared with later instances of data collection.

#### Mental health disorders

We explored adults ever diagnosed with any mental health problem as self-reported, identified through the mental health questionnaire. In 2016, as part of the online follow-up assessment, UK Biobank participants with a working email address were invited to complete an on-line mental health self-assessment questionnaire (category ID: 136). Participants were asked ‘have you been diagnosed with one or more of the following mental health problems by a professional, even if you don’t have it currently?’ (field ID: 20544). Participants were offered a set of options to choose between and were able to select multiple answers. The responses were coded as ‘social anxiety or social phobia = 1’, ‘schizophrenia = 2’, ‘any other type of psychosis or psychotic illness = 3’, ‘a personality disorder = 4’, ‘any other phobia (e.g. disabling fear of heights or spiders) = 5’, ‘panic attacks = 6’, ‘obsessive compulsive disorder = 7’, ‘mania, hypomania, bipolar or manic-depression = 10’, ‘depression = 11’, ‘bulimia nervosa = 12’, ‘psychological over-eating or binge-eating = 13’, ‘autism, asperger’s or autistic spectrum disorder = 14’, ‘anxiety, nerves or generalized anxiety disorder = 15’, ‘anorexia nervosa = 16’, ‘agoraphobia = 17’, ‘attention deficit or attention deficit and hyperactivity disorder = 18’, ‘prefer not to answer (group A) = −818’ and ‘prefer not to answer (group B) = −819’. All the participants who responded ‘prefer not to answer’ were excluded as mentioned before. For analytical feasibility, we categorised the 16 mental health problems into four groups: (a) neurotic and stress disorders (NSDs): social anxiety/social phobia, anxiety/nerves/generalised anxiety disorder, any other phobia (e.g. disabling fear of heights or spiders), panic attacks, agoraphobia and obsessive compulsive disorder; (b) mood disorders (MDs): mania/hypomania/bipolar/manic depression and depression; (c) eating disorders (EDs): anorexia nervosa, bulimia nervosa and psychological over-eating/binge-eating; (d) other mental health disorders (OMDs): schizophrenia, any other type of psychosis/psychotic illness, a personality disorder, autism/asperger’s/autistic spectrum disorder and attention deficit/attention deficit and hyperactivity disorder. Except OMDs, all other three categories were created according to the ICD 10 protocol. The ‘OMDs’ category was created to facilitate analysis and keeping in mind the widely acknowledged overlapping nature of mental health problems [9]. We recoded participant’s response into dichotomous variables: ‘yes = 1’ and ‘no = 0’. Participants never diagnosed of any mental health problems were considered as relatively healthy and were categorised as ‘reference’ in the study.

### Ascertainment of covariates

#### Sociodemographic characteristics (sex, age, educational status, townsend deprivation index, body mass index)

The variable sex was acquired from the central registry of the NHS at recruitment and was also updated on self-reporting. The age variable was extracted based on the participants’ date of birth and the participants’ attendance date for examination at the initial assessment center. The educational status was recoded into ‘college or university degree’ = 1 and ‘others’ = 0; ‘none of the above’, ‘prefer not to answer’ and ‘system missing’ were recorded into the missing value. The townsend deprivation index (TDI) variable was considered to assess sociodemographic deprivation at the area level. A TDI score of each participant was calculated based on residential post code and preceding national census output [22], those with negative values indicate relative affluence and the lower the number the more advantaged. Body mass index (BMI) characteristics of working individuals have been reported to influence job satisfaction, as lower BMI has been observed to be associated with higher job satisfaction [23]. BMI was calculated by using the equation: BMI = weight (kg)/height (m)^2^, and was further categorised according to the World Health Organization (WHO) classification into four groups [24]; underweight (<18.5 kg/m^2^), normal weight (18.5–24.9 kg/m^2^), overweight (25.0–29.9 kg/m^2^), and obese (⩾30.0 kg/m^2^). Covariates were all measured at baseline.

#### Physical activity

Physical activity information was taken into account, as a physically active lifestyle has been observed to help increase satisfaction [12]. Information on physical activity was extracted from the touchscreen-based questions by calculating the metabolic equivalent (MET) score, taking into account the weekly frequency and duration (in minutes) of walking, moderate physical activity and vigorous physical activity. Initially, extreme outliers were excluded (defined as having a *z*-score out of the range of ±3.29 for the duration of walking, duration of moderate physical activity and duration of vigorous physical activity). The MET score (minutes/week) was then derived using the coefficients for each category obtained from the international physical activity questionnaire short form, by using the formula: MET score = [(number of days/week of walking 10+ minutes × duration of walking × 3.3) + (number of days/week of moderate physical activity 10+ minutes × duration of moderate physical activity × 4.0) + (number of days/week of vigorous physical activity 10+ minutes × duration of vigorous physical activity × 8)] [25].

#### The personality trait neuroticism

The neuroticism score was calculated based on the participants’ response to 12 questions from the Eysenck personality inventory neuroticism scale (EPIN-R): (1) ‘does your mood often go up and down?’; (2) ‘do you ever feel “just miserable” for no reason?’; (3) ‘are you an irritable person?’; (4) ‘are your feelings easily hurt?’; (5) ‘do you often feel “fed up”?’; (6) ‘would you call yourself a nervous person?’; (7) ‘are you a worrier?’; (8) ‘would you call yourself tense or “highly strung”?’; (9) ‘do you worry too long after an embarrassing experience?’; (10) ‘do you suffer from “nerves”?’; (11) ‘do you often feel lonely?’; (12) ‘are you often troubled by feelings of guilt?’. The score ranged from 0 to 12, where a higher score represents a higher level of neuroticism [26].

#### Occupational related variables (work hours)

Work hours was defined as ‘length (hours) of working week in job’ with the question ‘in a typical week, how many hours do you spend at work? (do not include hours travelling to and from work)’. If the participant had more than one ‘current job’ then they were instructed to answer the question for the main job only. Considering the variable as continuous, a higher value represented more working hours per week. ‘Do not know’, ‘prefer not to answer’ and ‘system missing’ were considered as missing values.

### Statistical analyses

Sample characteristics were summarised using frequencies, proportions and mean with standard deviation. We determined the regression coefficient (*β*) and 95% confidence intervals (CIs) calculated by linear regressions to investigate the ordinal variable ‘job satisfaction’ and ‘job tenure’ distribution among the people with diagnosed mental health problems compared with relatively healthy people (reference) in the UK Biobank population. Both crude and covariate adjusted analyses were conducted. Adjusted analyses, to assess the influence of different covariates that might confound or mediate differences in the outcome, were conducted in two steps. In the first step, the analyses were adjusted for age, sex, TDI, BMI, education, physical activity and work hours. In the second and final step, the analyses model was additionally adjusted for neuroticism to account for the influence of personality. The results for both crude and adjusted models are presented in the manuscript. A *P* value of less than 0.05 was considered significant. For all the analysis, missing data were excluded and the percentage is reported in Supplemental Table I. SPSS software (IBM SPSS Statistics version 26) was used for statistical analysis.

## Results

We undertook initial analyses on 108,711 participants from the UK Biobank who responded to the employment questionnaire (Figure 1). Table I shows the outcome of unadjustewd analyses comparing individuals with and without mental health disorders. Higher unemployment rates were observed among people with mental health disorders in the four main categories investigated, that is, OMDs, 5% (*n*=52); EDs, 2.2% (*n*=33); MDs, 2.1% (*n*=519) and NSDs, 2.1% (*n*=410) compared with people without mental health disorders 1.5% (*n*=1112). Among different mental health categories, individuals with OMDs seemed to be mostly affected as compared with reference and other mental health categories, they showed the highest percentage for the items ‘unable to work due to sickness/disability’ 20.6% (*n*=215), ‘doing unpaid/voluntary work’ 1.5% (*n*=16) and ‘full/part-time student’ 0.8% (*n*=8) and are least represented in paid or self-employment, 67.4% (*n*=702). Moreover, individuals with EDs showed the highest percentage in category ‘looking after home and/family’ 6.8% (*n*=104).

**Table I.** Employment status of UK Biobank population (total *N*=108,711) who responded to the mental health questionnaire.

	Ref (*N*=72,988)	NSDs (*N*=19,762)	MDs (*N*=24,882)	EDs (*N*=1534)	OMDs (*N*=1042)
In paid employment/self-employed	67,618 (92.6)	17,215 (87.1)	21,558 (86.6)	1248 (81.4)	702 (67.4)
Looking after home and/family	2702 (3.7)	858 (4.3)	1090 (4.4)	104 (6.8)	49 (4.7)
Unable to work because of sickness/disability	729 (1)	1018 (5.2)	1358 (5.5)	120 (7.8)	215 (20.6)
Unemployed	1112 (1.5)	410 (2.1)	519 (2.1)	33 (2.2)	52 (5)
Doing unpaid/voluntary work	592 (0.8)	175 (0.9)	219 (0.9)	18 (1.2)	16 (1.5)
Full/part-time student	235 (0.3)	86 (0.4)	138 (0.6)	11 (0.7)	8 (0.8)

Values are expressed as *N* (%).

NSDs: neurotic and stress disorders; MDs: mood disorders; EDs: eating disorders; OMDs: other mental health disorders.

Further analyses were undertaken on a sample of 34,808 individuals who were considered as properly employed according to the baseline assessment employment status questionnaire (Figure 1). Sociodemographic and occupational characteristics are summarised in Table II. The mean age of the participants in the mental health disorders categories at recruitment was comparable with the mean age of the reference group. Women were more likely to show mental health problems compared with men. Individuals in the EDs group showed the highest gender dependent discrepancy. Individuals with reported diagnoses falling in the NSDs and MDs categories were found to be less educated and tend to be more obese compared with the reference group. Moreover, individuals with reported mental disorders were less deprived, were more likely to show signs of neuroticism, showed a similar physical activity profile and reported working fewer hours per week compared with individuals without mental health problems. Furthermore, individuals in different categories of mental health disorders were observed to be significantly (*P*<0.001) less satisfied with the job and have significantly (*P*<0.001) shorter job tenure compared with the reference category. Individuals in the OMDs group seemed to be least satisfied with their job, 4.1 (±1.1) and had shortest job tenure, 9.1 (±10.8) years compared with other mental health disorders and reference categories (Table II).

**Table II.** Sociodemographic and occupational profile of UK Biobank participants (total *N*=34,808) who reported being properly employed.

	Ref (*N*=23,806)	NSDs (*N*=5983)	MDs (*N*=7650)	EDs (*N*=469)	OMDs (*N*=236)
Age	53.4 ± 7.2	52.7 ± 6.9	52.3 ± 6.9	51.3 ± 6.4	51.5 ± 6.8
Sex					
Men	11,701 (49.2)	1946 (32.5)	2388 (31.2)	39 (8.3)	108 (45.8)
Women	12,105 (50.8)	4037 (67.5)	5262 (68.8)	430 (91.7)	128 (54.2)
Education					
College/university	11,695 (50.3)	2695 (45)	3598 (47)	262 (55.9)	134 (56.8)
Other	11,841 (49.7)	3288 (55)	4052 (53)	207 (44.1)	102 (43.2)
TDI	−1.4 ± 2.7	−1.2 ± 2.8	−1.1 ± 2.8	−0.7 ± 2.9	−0.3 ± 3.1
BMI	26.6 ± 4.4	26.9 ± 4.9	27.3 ± 5.2	26.5 ± 7.3	27.4 ± 5.1
Physical activity (MET score)	2248.4 ± 2182.7	2162.7 ± 2122.5	2155.5 ± 2177.0	2290.7 ± 2154.3	1976.0 ± 2227.9
Neuroticism	3.2 ± 2.8	5.9 ± 3.3	5.8 ± 3.3	6.1 ± 3.3	6.1 ± 3.5
Work hours (per week)	35.2 ± 13.1	33.5 ± 12.4	33.5 ±12.5	33.7 ± 12.8	32.0 ± 11.8
Job satisfaction	4.4 ± 0.8	4.2 ± 1.0	4.2 ± 1.0	4.2 ± 1.0	4.1 ± 1.1
Job tenure (years)	12.9 ± 11.4	11.7 ± 11.2	11.0 ± 11.0	9.6 ± 10.5	9.1 ± 10.8

Values are expressed as *N* (%) or mean ± standard deviation.

TDI: Townsend deprivation index; BMI: body mass index; NSDs: neurotic and stress disorders; MDs: mood disorders; EDs: eating disorders; OMDs: other mental health disorders.; MET: metabolic equivalent.

An in-depth analysis was performed studying the association between job satisfaction, job tenure and mental health problems in adjusted calculations, as displayed in Table III. Individuals with mental health disorders in all four categories showed significantly lower job satisfaction, an effect that was seen when correcting for multiple covariates including age, sex, TDI, BMI, education, physical activity and work hours (*P*<0.001). Compared with individuals with no mental health diagnosis, individuals in the OMDs group showed the highest deduction of job satisfaction followed by subjects in the EDs, MDs and NSDs groups, respectively. Finally, the analyses were additionally adjusted for neuroticism and it appeared to have a strong impact on the association between job satisfaction and different mental diseases, as observed in Table III. The association remained only significant for individuals with MDs (β −0.04, 95% CI −0.071 to −0.014) after correcting for neuroticism.

**Table III.** Regression analysis of job satisfaction and job tenure among individuals with mental health problems (NSDs, *n*=5983; MDs, *n*=7650; EDs, *n*=469; OMDs, *n*=236) and individuals without any mental health problems (Ref, *n*=23,806) in UK Biobank cohort who reported being properly employed.

	NSDs		MDs		EDs		OMDs	
	*β* (95% CI)	*R* ^2^	*β* (95% CI)	*R* ^2^	*β* (95% CI)	*R* ^2^	*β* (95% CI)	*R* ^2^
Job satisfaction								
Crude	−0.22*** (−0.24 to −0.19)	0.01	−0.24*** (−0.26 to −0.22)	0.01	−0.22*** (−0.30 to −0.15)	0.00	−0.36*** (−0.47 to −0.25)	0.00
Adjusted-multivariate^a^	−0.22*** (−0.24 to −0.19)	0.03	−0.23*** (−0.26 to −0.21)	0.03	−0.24*** (−0.33 to −0.16)	0.02	−0.28*** (−0.40 to −0.16)	0.02
Adjusted-multivariate^a^ and neuroticism	−0.03 (−0.06 to 0.00)	0.92	−0.04** (−0.07 to −0.01)	0.99	−0.06 (−0.16 to 0.03)	0.82	−0.05 (−0.18 to 0.09)	0.81
Job tenure								
Crude	−1.22*** (−1.54 to −0.89)	0.00	−1.86*** (−2.15 to −1.57)	0.00	−3.27*** (−4.32 to −2.23)	0.00	−3.83*** (−5.29 to −2.36)	0.00
Adjusted-multivariate^a^	−0.68*** (−1.04 to −0.33)	0.73	−1.00*** (−1.32 to −0.68)	0.74	−2.09*** (−3.22 to −0.95)	0.75	−2.32** (−3.89 to −0.74)	0.75
Adjusted-multivariate^a^ and neuroticism	−0.66** (−1.07 to −0.25)	0.74	−1.19*** (−1.56 to −0.82)	0.75	−1.83** (−3.07 to −0.58)	0.76	−2.44** (−4.23 to −0.66)	0.76

a Multivariate: age, sex, Townsend deprivation index (TDI), body mass index (BMI), education, physical activity and work hours; NSDs: neurotic and stress disorders; MDs: mood disorders; EDs: eating disorders; OMDs: other mental health disorders.

* *P*<0.05, **P<0.01, ***P<0.001.

To investigate the relationship of job tenure with mental health disorders, we performed regression analyses (Table III). Mental health problems were consistently and significantly associated with lower job tenure in all models throughout all categories of mental health disorders. The strong effect remained even after adding neuroticism to the adjusted model. Individuals in the OMDs group showed the highest deduction of job tenure followed by individuals in the categories EDs, MDs and NSDs, respectively.

## Discussion

To the best of our knowledge, this study is the largest cohort data analysis that has investigated the association of job satisfaction and job tenure with self-reported physician posed diagnosed mental health disorders in a comprehensive European sample. We found that people with a diagnosis of different types of mental health disorders as experienced during lifetime show poor job satisfaction and lower job tenure. Both relationships were found to be independent of age, sex, TDI, BMI, education, physical activity and working hours. Additional adjustments with the personality trait neuroticism revealed a strong and independent impact of neuroticism specifically on job satisfaction, which was not observed with job tenure.

Our results show that diagnosed mental health disorders experienced during lifetime are associated with lower job satisfaction. The findings are based on comparative investigations of job satisfaction between individuals with and without mental health illness, addressing 16 mental health conditions categorised in four major groups (NSDs, MDs, EDs and OMDs) in a large European sample. Our study covers a broad spectrum of mental health conditions, in contrast to earlier studies [1, 2, 6, 7], which investigated only highly prevalent disorders such as anxiety, depression and burn-out/stress. In addition to confirming associations of eight illnesses falling in the categories NSDs and MDs with lower job satisfaction, we report for the first time that diagnoses belonging to the categories EDs and OMDs are also strongly associated with job satisfaction. Previous reports [1, 2, 6, 7] are mostly based on self-reported conditions and studied mainly isolated outcomes in smaller samples such as overall physical and mental health [1, 2, 6], and relationship to meaningful work [7]. Our study allows the direct association of several mental health conditions with job satisfaction within one large cohort, thus avoiding a potentially high heterogeneity of data that may be abundant in, for example, meta-analysis approaches, which may limit the explanatory power [27]. Moreover, use of self-reported physician posed diagnoses helped us to avoid reporting bias arising from very subjective, temporary evaluations of the individual’s health state.

We observed that individuals in the OMDs group have the relatively strongest association with job satisfaction, which comprises diagnoses that may be linked to intellectual disability such as disorders belonging to the asperger’s or autistic spectrum disorder and attention deficit hyperactivity disorder. However, earlier studies detected higher job satisfaction in individuals with intellectual disability [8]. The different outcome needs to be interpreted with caution, due to the heterogeneity of the subgroup in our study that also included other diagnoses such as schizophrenia and personality disorder, that are related to job dissatisfaction [4]. Moreover, individuals with intellectual disability are reported to have an insufficient and improper concept of job satisfaction [28], as their perception of work could be related to the opportunity to remain social and this could be translated to the overly positive characterisation of work.

We found that job tenure was inversely associated with mental health conditions. Here, we confirm previous small scale (*N*~60–326) [3, 15–20] studies, adding a wider range of investigated diagnoses. We observed that individuals with mental health conditions show shorter job tenure compared to healthy individuals. Earlier studies performed mainly survival analysis predictions [15, 18, 19] or studied job termination in different employment programmes [3, 4, 16, 17, 20]. The literature on job tenure in relation to various mental health categories is scarce and has mostly focused on limited diagnosed conditions such as general psychiatric disability [17, 19], schizophrenia, personality and mood disorders [3, 4, 15, 16, 18, 20]. Thus our study is unique regarding the broad spectrum of mental health diagnoses investigated.

The association between lower job tenure and mental health disorder was highest among the individuals in the OMDs group, followed by individuals in the categories EDs, MDs and NSDs. This outcome may be a result of the aspect that individuals with schizophrenia or personality disorders were included in the OMDs group [3, 4, 15, 16, 18, 20]. As noted in our unadjusted analyses, individuals in the OMDs category most often responded with ‘unable to work because of sickness/ disability’ and fell most often in the lowest paid employment section compared with other categories, which may also explain the shortest job tenure of this group. These results have to be interpreted with caution due to the heterogeneity of the data in this group and the small sample size.

We found a strong association of neuroticism with job satisfaction throughout different mental health conditions in line with previous evidence [10, 13, 14, 29]. A recent meta-analysis reported that the neurotic personality trait is the strongest predictor of job satisfaction among the big five personality traits [11]. Furthermore, a positive relationship between job satisfaction and the absence of negative affect in life has been shown [30]. Thus the abundance of neuroticism in individuals with mental health problems is likely to be one of the main factors for lower job satisfaction in line with previous findings [11, 12, 30]. One exception to this is MDs, for which strong associations between job satisfaction and mental disorders were found to be independent of the neuroticism score. This is in line with observations in longitudinal and genetic analyses showing that neuroticism strongly reflects the liability to depression [31]. Hitherto only a few studies with a small sample size (e.g. Fortin et al. [5], *N*=82) investigated personality factors with employment, and found that negative personality traits were associated with a delay of job acquisition, but not with job retention or tenure. According to our results neuroticism does not influence job tenure, which confirms previous findings [5].

Interestingly, we observed that individuals with mental health disorders appeared to be less deprived and to work fewer hours per week. This may be explained by flexible work schedules and accommodation such as privileges provided by employers to subjects with psychiatric disabilities [4]. The amount of physical activity was comparable in individuals with and without mental health conditions. Considering that physical activity may help in improving job satisfaction [12], we may assume that individuals with mental health disorders may benefit from being more physically active; however, further investigation in this domain is recommended to draw reliable conclusions.

The strength of the present study is the high statistical power due to the large sample size of the UK Biobank cohort. The availability of the detailed information covering lifestyle, sociodemographic factors and work-related parameters made it possible to correct for a considerable amount of potential confounding factors, and allowed us to draw conclusions for a general European population. Furthermore, the study includes a large panel of mental health disorders covering 16 diagnoses, in contrast to most previous studies that included only three to four prevalent conditions. However, certain limitations need to be mentioned. First, the time difference between the collection of job-related data and mental health data is a limitation in this cross-sectional analysis. It cannot be excluded that some of the individuals may have had some of the mental health-related diagnoses in the 6 years after the baseline investigation. However, it can confidently be assumed that the vast majority of events have happened in the period up to the baseline in the cohort of older individuals aged from 45 years and upwards, as the covered diagnoses have their peak usually in younger years, and the majority of collected person-years lie in the time frame up to the baseline investigation. It can be furthermore assumed that mental health disorders tend to be chronic conditions which often lead to a long-time history with delayed diagnosis, as mentioned by us in the paper [32]. Second, the category OMDs contained a wide range of different mental health diagnoses. We were not able to subcategorise individuals into groups of isolated diagnoses due to the strong overlapping nature of mental health disorders [9]. Third, we could have considered additional confounders in the analyses such as job-environment related factors because job tenure relates to job environmental factors [3] or factors related to subjective wellbeing as they are also associated with job satisfaction [30]. However, to keep our analysis as specific as possible and to avoid an overload of the models we decided to include factors most strongly related to job satisfaction. Finally, the study is based on cross-sectional data which does not allow us to infer causality. However, we clearly observed that mental illness experienced during lifetime independently associates with job satisfaction and tenure. As thoroughly investigated in a recent meta-analysis [1], employees with mental health issues may suffer in their waking hours at work for a long time, leading them to experience a lowering feeling of self-worth, feeling unfulfilled for long periods of working days or actual dissatisfaction. Moreover, job-related components are different in each job facet and employees have different expectations from the organisation when working in these facets [1, 3]. These differences are important and may need to be addressed properly when considering the job satisfaction or job tenure of the employees. However, it can be hypothesised that a proper management of mental illness in the working place may notably improve the employment situation of affected individuals.

## Conclusions

The present study demonstrates a strong link between mental health conditions, job dissatisfaction and job tenure. Neuroticism was strongly linked to job satisfaction but was not associated with job tenure suggesting a differential role of this personality trait. Overall, our findings clarify the complex relationship of mental health with job satisfaction and job tenure. A detailed understanding of these relationships and the possible relevant interacting factors such as personality traits are important to design measures such as human resources training and services to improve working life participation of individuals with mental health issues.

## Supplemental Material

sj-docx-1-sjp-10.1177_14034948221119639 – Supplemental material for Job satisfaction and job tenure of people with mental health disorders: a UK Biobank cohort study Click here for additional data file. Supplemental material, sj-docx-1-sjp-10.1177_14034948221119639 for Job satisfaction and job tenure of people with mental health disorders: a UK Biobank cohort study by Salahuddin Mohammad, Maud Miguet, Gull Rukh, Helgi B. Schiöth and Jessica Mwinyi in Scandinavian Journal of Public Health
