# Supplementary material for: Job satisfaction and job tenure of people with mental health disorders: a UK Biobank cohort study
Source: Scand J Public Health. 2022 Aug 25;51(8):1248–57. doi: 10.1177/14034948221119639 (PMC10642223; doi:10.1177/14034948221119639)
Supplement: sj-docx-1-sjp-10.1177_14034948221119639 – Supplemental material for Job satisfaction and job tenure of people with mental health disorders: a UK Biobank cohort study [file sj-docx-1-sjp-10.1177_14034948221119639.docx]

**Supplementary Materials:**

**Supplementary Table S1.** Number and percentage of missing values for outcome variables.

|  | **n Valid** | **n Missing** | **% Missing** |
| --- | --- | --- | --- |
| *Employment status* | 108 711 | 0 | 0 |
| *Age* | 34 808 | 0 | 0 |
| *Sex* | 34 808 | 0 | 0 |
| *Education* | 34 808 | 0 | 0 |
| *TDI* | 34 733 | 75 | 0.2 |
| *BMI* | 34 706 | 102 | 0.3 |
| *Physical Activity (MET Score)* | 28 292 | 6 516 | 18.7 |
| *Neuroticism* | 29 706 | 5 102 | 14.7 |
| *Work Hours (Per Week)* | 34 322 | 486 | 1.4 |
| *Job Satisfaction* | 34 808 | 0 | 0 |
| *Job Tenure (Years)* | 34 712 | 96 | 0.3 |
